# Grounding co‐writing: An analysis of the theoretical basis of a new approach in mental health care

**DOI:** 10.1111/jpm.12835

**Published:** 2022-04-28

**Authors:** Elena Faccio, Raffaella Pocobello, Roberto Vitelli, Giovanni Stanghellini

**Affiliations:** ^1^ Department of Philosophy, Sociology, Education and Applied Psychology Padua Italy; ^2^ Institute of Cognitive Science and Technology of the National Research Council (ISTC‐CNR) Rome Italy; ^3^ Department of Neuroscience and Reproductive and Odontostomatological Sciences University of Naples “Federico II” Napoli Italy; ^4^ Department of Psychological Sciences Health and Territory, "G. d'Annunzio" University Chieti Italy; ^5^ Department of Psychological Sciences Adjuncto Universidad "Diego Portales" Santiago Chile

**Keywords:** co‐writing, mental health care system, narratives, phenomenology, power, recovery

## Abstract

This contribution aims to highlight the theoretical and epistemological premises of the co‐writing experience, a practice where a clinician and a patient are mutually engaged in jointly or collaboratively writing a narrative related to the patient’s experience. Unlike a typical set of therapeutic techniques, co‐writing is based on sharing perspectives and meanings about the experience of crisis, recovery, and the therapeutic process. The paper identifies and briefly describes four non‐clinical epistemological paradigms on which it is grounded: ethnography, values‐based practice, narrative care, and phenomenology. Although they differ in several ways, at the same time, they seem to share some common features that the paper investigates and comments.

For clinicians, nurses, researchers and Mental Health Service managers, attention to the users and to the improvement of their active roles represents not only a strategy for the empowerment of results, but also the access door to a different perspective which relies on a renewed conceptualization of the mental disease nature that may lead to overcoming the epistemic asymmetry between the ‘expert’ and the ‘other’ in favor of intersubjective dialogue.

## 1 INTRODUCTION

The idea that the relationship between mental health professionals and patients may be characterized as a form of collaboration has certainly marked a jump forward in how we conceive of and treat mental suffering. Among the various forms of collaboration available in the literature, this paper presents and discusses the premises of a very particular one: *co*‐*writing*, a specific practice where a clinician and a patient are mutually engaged in jointly or collaboratively writing a narrative related to the patient's experience. For clinicians, nurses, researchers and Mental Health Service managers, attention to the users and the improvement of their active roles represents not only a strategy for the empowerment of results but also the access door to a different perspective in the approach to mental health treatment and research, aimed at balancing the power relation between clinical staff and patients. Co‐writing is a method that aims to minimize injustice in mental health by integrating everyone's voices into a balanced narrative (Fricker, 2007).

Writing jointly the patient's story, or supporting him/her to do it, including medical records and all documents concerning his/her mental health, means radically challenging the more often adopted settings and practices where the expert manages the power of defining the mental health condition and treatment planning and the patient merely undergoes it. Unlike a typical set of therapeutic techniques, co‐writing is based on sharing perspectives and meanings about the patient's personal experience of crisis, recovery and therapeutic process. It represents an innovative approach that may foster new responses to dilemmas faced by patients in the mental healthcare system. As it is clear, this new approach also carries new responsibilities for therapists, mental health professionals and nurses. Given the complexity of the topic, we would like to develop this complex issue throughout three papers, intertwined according to sequential logic.

This first one is about the *theoretical and epistemological premises* of the co‐writing experience. It identifies and briefly describes four non‐clinical epistemological paradigms on which co‐writing is grounded: ethnography, value‐based practice, narrative care and phenomenology. Although they differ in several ways, at the same time, they seem to share some common features that we aim to investigate and comment on.

A successive paper will consider *purposes and forms of co*‐*writing* trying to systematize the possible objectives and potential benefits for professionals, nurses and patients. It will also articulate the forms and techniques of the collaborative experiences. A final paper will consider the *ethical implication of the collaborative approach thoroughly*. Stories are vital tools for professionals asking ethical questions about doing what is right in the human‐universe‐health process. They are living entities of community that may be used for research, education and practice since they provide notions for further ethical thinking and implications for human regard from a clinical and nursing theoretical perspective.

Attention to personal stories and needs becomes particularly important in a historical and cultural background characterized by growing social and political pressures towards restoring old practices of social control and custodialism (Antonio Iudici et al., 2022). This phenomenon has arisen an opposite pressure from other "subordinate" social agents who claim the right to challenge the dominant knowledge about mental illness and replace it with alternative knowledge, built not by mental health professionals but by the users themselves. In the recent past, the appearance on the scene of patient associations or patients’ families has only partially contributed to the progress of the clinical disciplines, leading instead sometimes to an acute and non‐productive conflict between the parties involved (Jugessur & Iles, 2009). All this makes it necessary to implement collaboration practices between professionals and users in view not only of the construction of effective treatment paths but also of the formulation of knowledge on mental discomfort and illness that arises from the dialogue between the various stakeholders and leads in the direction of a synthesis.

This is crucial for mental health nursing roles since they will be required to know the new forms of the collaborative approach, but also, they will be called upon to update themselves on their implementation by attending training courses about how to apply them with respect to their specific role. We think this paper may enhance reflexivity about clinical practice and offer suggestions about how mental health nurses may promote patient empowerment and active roles. Our objectives are to propose recommendations based on the scientific literature to help maximize the benefits and minimize the risks of such involvement.

## 2 EPISTEMOLOGICAL AND THEORETICAL PREMISES

Towards the end of the 1970s, certain approaches to the study of human existence spread in psychology and the human sciences. Despite their diversity, they shared the assumption that the unit of analysis of mental phenomena should not be an individual isolated from their context but rather the intrinsic *relatedness* of a person in interaction with others (Gergen, 2009; Spinelli, 2015).

According to the complexity paradigm, knowledge is always a perspective relative to the observer's point of view with respect to the observed (Morin, 1992).

Several interpretations are possible and can legitimately coexist when human behaviour is concerned, including the perspective of the *object* observed alongside the perspective of the observing subject. Our knowledge of human behaviour should be embedded and situated within the scientific community. The human sciences have a mainly *rhetorical* foundation since our understanding of human phenomena is a more or less shared form of knowledge negotiated within the scientific community (Kuhn, 1996; Latour & Woolgar, 1979). Scientific methods, far from guaranteeing *objective* knowledge about the human being, cut out the reality in ways arising from the specific methodology adopted. Knowledge of human behaviour should take as its object the person as a social agent in their relational world and have as one of its epistemological considerations the integration of competing theories and perspectives within the scientific community (Lobo et al., 2018).

In harmony with these assumptions, a basic principle has gained more and more value. This can be formulated as follows: if we want to gain knowledge of another human being, that human being's voice should have the right to be listened to and acknowledged (Mascolo & Kallio, 2020). Epistemic asymmetry between a person as an object of knowledge and another person as a knowing subject should be avoided or reduced to a minimum. These changes may initiate a radical transformation of mental healthcare practice, which would only be possible thanks to a renewed conceptualization and epistemology of the mental disease nature. The overall principle leading this revolution is to embed the practice of care within a relational context that tries to include all the involved actors. We may call this approach *ecological*, as ecology is the science that studies the dynamics of a wide array of interactions between the different levels of a living system (Bateson, 1972).

The translation of these assumptions into practices aimed at mental health care has led to a radical revision of the clinical encounter. Interviewing a patient can no longer be compared to two people assembling a puzzle, where the patient has the pieces and the interviewer the image of the completed design (Othmer & Othmer, 1994). Mental health professionals are no longer conceptualized merely as *experts* on *puzzled* people or as objective observers holding the power and authority of defining the reality of the patient's troubled experiences. Instead, in the context of the therapeutic dialogue, the clinician is considered as a facilitator and collaborative partner in the unfolding of patients’ stories and in the search for new meanings regarding their suffering (Vitelli, 2018). Moreover, clinicians and patients have an opportunity to reexamine their experiences and co‐construct a narrative that is more consistent with their values and purposes and the way they develop in the course of therapeutic dialogues. Collaborative writing is one of the effects of this innovative way of understanding the relationship between clinician and patient; the objectives it pursues, including the therapeutic ones, can be more fully grasped in light of the specific theoretically driven research methodologies to which we give space below.

In the following sections, we succinctly describe four main paradigms contributing to this *ecological* approach to mental health care, all of which are coherent with the practice of co‐writing: ethnography, value‐based practice, narrative care and phenomenology.

### 2.1 Ethnography

Ethnography (from the Greek ἔθνος [ethnos; “folk, people, a nation”] and γράφω [grapho; “I write”]) means a lengthy written description that creates awareness and understanding of social patterns in a specific cultural context. It relies heavily on researchers participating in a setting together with the people being studied. It seeks to document, in detail, patterns of social interaction and participants’ perspectives with the goal of understanding these in their local contexts. Its ethical core is that no human phenomenon or behaviour is necessarily meaningless or crazy; rather, it may reveal itself as meaningful when considered adequately within its socio‐cultural context. More generally, in the context of mental health care, this approach values the clinical encounter as a sort of meeting of cultures, including those of the patient and the clinician.

Writing is an essential component of ethnographic research, as the researchers combine other data collection methods with their own observations written in a personal notebook. The researchers’ subjective perspectives on their experiences are considered an integral part of the research and are valued as opportunities for reflexivity. Within ethnographic research, the particular type of collaborative writing called *collaborative and relational autoethnography* came to life as a method of investigation that connects qualitative research and self‐writing guided by the research leader. Researchers play the role of recipients and co‐authors, help organize writings, compose stories themselves and support participants (users, families and health professionals) in writing experiences related to the mental health field. This approach allows researchers to facilitate the expression of other people's stories in an evocative way; the conversation between the researcher and the natives makes possible a shared narration and its transcription (De Serpa et al., 2019). Autoethnography is a contemporary qualitative research methodology that works with the idea of a provisional and contingent historical truth (or *version*; Short et al., 2013).

In De Serpa's study (De Serpa et al., 2017), the authors address the benefits and complications of collaborative testimony and investigate how it includes the dimension of otherness, as the autoethnographic perspective consists of writing for and with the other, listening and working together. Self‐narration is valued as a way to gain access to a person's social relationships and cultural background in a back and forth movement that connects a personal point of view to a collective context (Ellis et al., 2015). The encounter between the native and the researcher (the encounter with “otherness”) that characterizes anthropological work so strongly has been considered as a prototype of all encounters with otherness, not least the clinical meeting (Clifford & Marcus, 1986; Faccio & Fovino, 2019).

The *other* exists as a single person, but also as a testimony of the cultural system he represents; so the encounter is also a means of getting to know an entire culture which, through one of its representatives, tiptoes into the encounter. The *other* enters the session with all their background, carrying a vision of reality (worldview) derived from socialization, and, with them, a whole community enters the interview room. Bizarre or meaningless behaviours become significant if the researchers themselves take on the gaze of the native community (see De Martino's critical ethnocentrism in Saunders, 1993). The other is encountered and known from a relational perspective. The writing of one's own story testifies to the intrinsic relationality of the ethnographical method: the writer always writes for someone—to tell them what the writer already has in mind (Bacigalupe, 1996).

The distance between the researcher and the other, traditionally positively valued in scientific research, is discarded in favour of intersubjective dialogue. The relationship promotes an exercise in self‐reflection on the part of those who produce knowledge, including considerations regarding the consequences that this knowledge brings to others (Adams et al., 2015).

An example is storytelling in relational autoethnography (Elli & Rawicki, 2013; Klevan et al., 2018), which can be conceived of as a relational game during which both the narrator and the listener are engaged in minimizing inconsistencies. Participants try to organize intersections and contradictions in a temporal continuum and incorporate these into a narrative that gives order and organization to events. These efforts are produced both by the person who tells the story and by the person who listens to someone else's story. Through the narrator, the bizarre and the improper are inserted into a meaningful plot and made logical in the listener's mind (Ellis et al., 2015). The listener is required to unfold the details of the narrator's daily actions and assume their roles as completely as possible in order to enter into the other's experience as if it were the listener's own experience (De Serpa et al., 2017; Ellis & Adams, 2014).

### 2.2 Value‐based practice

Value‐based practice (VBP), in its various forms, is a method whose aim is to recognize and integrate participants’ perspectives as part of the co‐production of a project, rather than imposing the carer's perspective. Beginning in the 1970s, in various fields, including economics and commerce and the reform of the justice system, public policies and public health services, practitioners began to think that users (or consumers) could be empowered as privileged witnesses, able to offer an *expert* vision of the service based on first‐person experience and able to take part in the delicate phase of adapting services to their needs. The rise of this movement, called *co*‐*production* in social care, was strongly linked with the disability movement and the mental health user movement. The statutory guidance for the Care Act 2014 (UK) defined “co‐production” as the situation where “a person influences the support and services received, or when groups of people get together to influence how services are designed, commissioned and delivered.” The concept of co‐production has become increasingly recognized in healthcare settings in recent years, and many of the core values of co‐production are now at the heart of government legislation that informs services (Co‐production and the Care Act, 2014). The co‐production process involves joint decision‐making and shared power between service users and professionals. Service users are here considered as equal partners in the design and evaluation of services rather than as mere recipients of a service (Pocobello et al, 2020). People who use the service are *hidden* resources, and any service, to be efficient, cannot ignore this resource (Boyle & Harris, 2009). Doing *with*, rather than doing *for*, ensures better recovery results for service users (New Economics Foundation [NEF], research commissioned by MIND), favouring improvements in their well‐being, self‐esteem, trust and social inclusion (Slay & Stephens, 2013). As matter of fact, still today more often inpatient and mental health services are power‐dominated hierarchical systems in which practitioners hold the role of sole actors in decision‐making processes related to patients’ life choices and justify their dominance in terms of their responsibility for care (Cott, 1997; Davies et al., 2006). Therefore, facilitating a shift in the distribution of power and a push towards co‐produced services can be particularly challenging in this context not least because of the impact of legal restrictions and entrenched role stereotypes (Lewis‐Morton et al., 2017; Reilly, 2013).

VBP is among the first authoritative examples of the co‐production of mental health services. As Sackett et al. stated some years ago, values are not synonymous with ethics, as, more generally, they encompass the “unique preferences, concerns, and expectations each patient brings to a clinical encounter” (Sackett et al.; 2000, p. 1). VBP is the theory and practice of effective healthcare decision‐making where different (and hence potentially conflicting) values are in play (Fulford, 2005). VBP, primarily a derivative of philosophical value theory, adds to the standard resources for diagnosis in psychiatry based on symptom recognition by offering a set of practical tools for working effectively in areas where clinical decision‐making depends not only on complex evidence (addressed by evidence‐based practice) but also on complex values (Fulford et al., 2005). The specific contributions of VBP include (a) raising awareness of the role of values even in categorical psychiatric diagnostic systems (e.g. the DSM), (b) providing a clear theoretical explanation for the relative prominence of values in psychiatric diagnostic classifications (derived from the relative complexity of human values in the areas with which psychiatry is concerned), and (c) the policy frameworks and training methods that have already been established for value‐based practice. VBP was first introduced into the work of the UK’s Department of Health through the joint programme between patients and professionals that led to the adoption of the Framework of Values by the National Institute for Mental Health in England (NIMHE & Department of Health, 2004). The NIMHE Framework of Values thus provides a robust policy platform for ensuring that value‐based and evidence‐based approaches underpin service development in all areas of mental health and social care.

Another example is the Strategy for Patient‐Oriented Research promoted in 2010 by the Canadian Institutes of Health Research (CIHR) to support user‐oriented research in Canada. The CIHR define this research as a continuum identified as improving the therapeutic path by including patients as partners and focusing on their priorities. It is carried out by multidisciplinary teams in collaboration with interested parties and aims to apply the knowledge generated in the process to improve health systems and practices. The CIHR define patients and informal caregivers, including family and friends, as experts in a health problem. Furthermore, their research involvement is expressed in terms of meaningful and active collaboration in governance, prioritization and the conduct of research and knowledge generation. Richards et al. (2020) argued that patients must be informed, empowered and considered as active partners in their health care, especially in defining the treatment plan, including the pharmacological component.

Within this framework, there is also the so‐called conceptual model of the Patient‐Centered Outcomes Research Institute (PCORI), which defines the collaboration process between users and medical staff as based on seven principles: trust, honesty, co‐learning, transparency, mutual relations, partnership and respect. The intent is to work with patients and their family members as co‐investigators for an extended period, allowing everyone to get to know each other and develop an informed awareness of the care process (Browne et al., 2020).

### 2.3 The narrative approach

The core concept of the narrative approach is that events, including symptoms, are part of a meaningful narrative. The reconstruction of this narrative has the power to become a healing story. Many authors have explored the relevance of linguistic aspects in the construction and deconstruction of identity and mental phenomena (Bang, 2009; Branco & Valsiner, 2010; Graham & Stephens, 1994). Whether our stance with respect to a *narratological view* is radical (Gergen, 1991) or moderate (Spence, 1982), it is undeniable that meanings are recreated through *re*‐*storying exercises*. The idea that the human being is engaged in narrating stories from which they draw awareness of their own self is not simply one normative ideal among others but rather “our only or ‘primary’ way of organizing our experience in time” (Chandler, 2000, p. 215). In this view, since the self is primarily a set of *narratives relating to the* identity, permanence and change may be conceptualized through the evolution of the configuration of reality produced by narratives about the self. Self‐writing is conceived within this approach as a real therapeutic re‐narration laboratory (Smorti, 2007) and supports the identity evolution process during therapy.

When writing about themselves, people re‐organize biographical events, making them consistent with the present, past and future, and integrate their thoughts and emotions (Faccio et al., 2019). Their interest in narrating is thus linked to the need to rethink past events and give them new meaning. Self‐narrations may help reconfigure critical events by giving a guiding reason around which the story is formed (a story goal), including important events that relate to this story goal, and putting the events in a sensible order (Gergen & Gergen, 1987, 1988). When writing stories, the writer must connect events and episodes and, when building links, must provide an internal meaning and coherence to what someone else (in a more external role) would consider illogical and absurd. For this reason, building stories is a way to justify emotions and feelings, making them rational, logical and appropriate (Bruner, 1990; Faccio, 2011). The act of narrating stories may be considered as a meaning‐making process where past events are not just described but also reconstructed and where stories are not just copies of reality but also reinterpretations. Bruner (1990) used the term *mimesis* to explain the centrality of authors’ subjective contributions in narrating stories: personal writing is always a metaphor, an interpretation of reality, related to meanings used by everyone when constructing their world. The common elements in any narration include (a) the sequencing of events (the plot that structures the events in a sensible order), (b) the contraposition between *real* facts and the writers’ interpretations (a crucial element in psychotherapy), and (c) the links between ordinary and extraordinary facts, behaviours and events. The first element represents all cultural manifestations shared by people and is immediately intelligible, while the second links personal occurrences and feelings (Bruner, 1990). Writing provides an opportunity to describe the reasons and intentions that can make a story understandable to others. The writing and re‐writing of personal stories in clinical context seem to help increasing a sense of control over the past: “once an experience has structure and meaning, it follows that the emotional effects of that experience are more manageable” (Pennebaker & Seagal, 1999, p. 1243). Among the many research contributions available on the subject, it is worth mentioning one of Smorti (Smorti et al., 2010), who evidenced that analysis of language in the narratives collected within psychiatric patients attending an autobiographical laboratory, showed how inpatients passed from a narrative that was more centred on the memory of the past to a narrative that was more similar to a conversation and enriched with "insight" terms and the use of verbs in the conjunctive form, consistently with the improvement that was observed in inpatients’ social functioning by the medical staff.

Painful events that are not structured into a narrative format may contribute to the continued rumination of negative thoughts and feelings, but co‐writing can help people manage the emotional distress that arises from not having placed their experiences into a narrative structure to make sense of it and contain stressful emotions (Cipolletta et al., 2019; Faccio et al., 2012; Mahoney, 1995).

Accordingly, research devoted to the effects of expressive writing on traumatic or stressful events and the psychological and psychophysiological mechanisms that become active when these events are translated into words (Pennebaker, 1997) revealed that when people write about their emotional upheaval, there is a marked improvement in their physical and mental health. Since positive benefits in health and behaviour have been found in people from different social classes and ethnic groups, writing can be considered an effective cross‐cultural tool (Dominguez et al., 1995; Spera et al., 1994). Moreover, several studies found that writing or talking about painful emotions can influence and improve biological factors (Pennebaker et al., 1988).

Building on these important theoretical and empirical bases, self‐writing has been strongly implemented in the practices of narrative medicine. This is to make the patient an active subject and involve them in decisions regarding health care. The assumption is that the narrative understanding of one's care path improves the patient–clinician relationship and contributes to optimizing the process of active adherence to therapy. In addition, the reading of the texts produced by the patient allows the clinician to capture and measure the patient's general competencies, communication skills, attitudes towards life and health problems (Pearson et al., 2008).

Accordingly, the narrative psychiatric approach emphasizes the potentiality of shaping lives through storytelling and learning from other's stories: rather than focusing only on finding the source of the problem, adopting this collaborative clinical approach, psychiatrists also help patients to put in frame their suffering and develop their sources of strength. By encouraging the patient to explore their personal narrative through questioning and storytelling, the clinician helps the patient participate in and discover how they construct meaning, how they view themselves, their values and who they want to be. These revelations, in turn, inform clinical decision‐making about what it is that ails them, how they'd like to treat it, and what recovery might look like (Hamkins, 2013).

### 2.4 Phenomenology

Phenomenology is a discipline that studies human lived experience and its pre‐reflexive conditions of possibility. Born at the end of the nineteenth century as a philosophical discipline, it has gained relevance in psychiatry, psychology and psychotherapy since the 1920s (Stanghellini et al., 2018). It may well be regarded as a foundational science not only for psychopathological knowledge but also for the theory and practice of mental health care. It provides an approach that captures human existence in all its dimensions, from self‐awareness and embodiment to spatiality, temporality, narrativity and intersubjectivity (including their implicit forms). Moreover, it offers a view that localizes mental disorder not in the hidden convolutions of the brain nor in the hidden corners of the patient's psyche but rather in their lived experience and relations with others.

The phenomenological approach implies a methodical suspension of our commonsensical assumptions about the shared world. Phenomenologists use the word *epochè* to address the cessation of prejudices. This involves suspending belief in something or, more precisely, not operating with some belief. It is a way to bracket our habit of considering consciousness and the world based on common sense, putting to one side the default natural science understanding of them and making a transition to a way of considering them based on things themselves (McKenna, 1997). It includes a cessation of both our obvious/common sense and our theoretical/scientific pre‐knowledge (e.g. ipothetic neurobiological basis of mental disorders and previously established diagnostic categories). This cessation exhibits a respect for the evidence of experience and, at the same time, orients itself towards seeking the grounds for that evidence. It enables the researcher/clinician to transpose themselves into fundamentally different ways of finding oneself in the world, presupposing—in their endeavour to understand the other—difference as well as resemblance between their ways of experiencing the world (Fuchs et al., 2019).

Phenomenology argues that the other can be understood if and only if the researcher/clinician is able to *reconstruct* the other's form of life within their *life world*. A life world is the province of reality inhabited by a given person, having its own *meaning structure* and a *style of subjective experience and action* determined by a given *pragmatic motive*. If we always have to consider the importance of the prelinguistic dimensions of consciousness as our initial relation to the world through our moods, affect and feelings, at the same time, the process of world disclosure prior to the language context is considered as always being rudimentary. We are the stories we tell ourselves and others, and *world projects* are nothing more than the sedimentation of inner‐life history into distinctive and highly individual *life themes* (Binswanger, 1928). As mental schemes, *themes* frame, direct and orient the way we feel and make sense of the world in which we live and the way we *move* ourselves within the intersubjective arena—in brief, our whole being‐in‐the‐world and our being‐with‐others (Vitelli, 2018).

The path to be followed in psychotherapeutic practices such as co‐writing is a diachronically established relationship based on circularity and reciprocity. The work to be carried out within the clinical encounter can be imagined as *co*‐*construction* work aimed at discovering the structural plot that founds the specific patient's *world project*, *the life themes* that enmesh their eventual abnormal experiences. More precisely, it should be considered as a specific methodology aimed at enabling clients to discover by themselves their idiosyncratic frames of reference or significance (i.e. the unrecognized presuppositions underlying their specific modalities of organizing their experiences in a meaningful way). At its base, there is the co‐creation and co‐habitation of a *therapy world* that is distinguishable from both the client's and the therapist's wider world experiences and rejects any unnecessary imbalances in the power aspects of the relationship (Spinelli, 2015).

Although most people are situated in a shared life world, there are several other life worlds, such as fantasy worlds, the dream world and what we may call here *psychopathological worlds*. Understanding another person requires the reconstruction of this person's life world, as their experiences and actions become meaningful if and only if they are posited within the life world they inhabit.

The supposition that the other lives in a world just like my own (i.e. they experience time, space, their own body, others, the materiality of objects, etc., just like I do) is often the source of serious misunderstandings. To understand the other, I need to acknowledge the *existential difference*—the individual autonomy—that separates me from the way of being in the world that characterizes them. Any forgetting of this difference will be an obstacle to understanding since these people live in a life world whose structure is (at least in part) different from my own. Achieving second‐order understanding thus requires me to set aside my pre‐reflexive, natural attitude (in which my first‐order understanding capacities are rooted) and approach the other's world as if I were exploring an unknown and alien country.

Four concepts in the phenomenological method seem to be relevant for supporting the practice of co‐writing in mental health care: *dialogue*, *attunement*, *recognition* and *intimacy*. Dialogue is the essential happening of language, not a mere exchange of information. Subjectivity is displaced, and something new about the interlocutors is revealed. Attunement is a modulation of the emotional field between myself and the other. It is also the capacity to coordinate my tempo with that of the other. Attunement, with its inter‐emotionality and inter‐temporality, is grounded in corporeality as a form of intercorporeality. Recognition is the epistemic and ethical capacity to acknowledge the alterity in myself and the other person. Self‐recognition is the acknowledgement of the pre‐individual elements not yet appropriated by myself, while other‐recognition is the acknowledgement of the other person as a fellow person to whom I attribute value, life and consciousness. Intimacy is an atmospheric experience of aloneness–togetherness and self‐ and other‐recognition; enveloped in an atmosphere of intimacy, I get in touch with myself via our becoming in touch with each other.

## 3 CONCLUSIONS

Co‐writing as a practice in which clinician and patient are mutually and collaboratively engaged in writing a narrative related to the patient's experience, story life, recovery or therapeutic process is today gaining special attention from the scientific community.

There is also an increasing emphasis on, and commitment to, valuing patients’ narratives in nursing practice and nurse education (Buckley et al., 2016; Phillips et al., 2016). Listening to the voices of those receiving care is just the beginning. The challenge is to use these narratives to improve practice and the patient experience, to explore the unintended consequences of communication between a nurse and a patient, as well as how the environment in which patients find themselves can relay important messages (Faccio, Aquili et al., 2021, Faccio, Author et al., 2021; Petty et al., 2018).

This paper has identified and briefly described four non‐clinical epistemological paradigms on which co‐writing is grounded: ethnography, value‐based practice, narrative care and phenomenology. Although they differ in several ways, at the same time, they seem to share some common features:

First, all of them seem to be guided by a fundamental ethical stance: the profound respect for lived experience as essential, legitimate and valid *knowledge*, where the co‐writing process elicits the discovery, emergence and reorganization of different *truths* (i.e. different modalities for framing one's own life experience). This fundamental ethical stance is in line with the recovery approach, where the role of lived experience is central to overcoming the rhetoric of suffering by defining a self apart from disorder and control (Jacobson & Greenley, 2001). Indeed, persons with lived experience of mental health issues within the recovery process are no longer confined to the role and position of a passive subject on whom clinicians operate based on their knowledge. On the contrary, they are considered as active actors leading their recovery journey.

Second, these paradigms intrinsically reject reductionism, especially the irreducibility of the mind to biology, acknowledging different forms of emergence. Indeed, the experience of mental health issues, the therapeutic encounter and the recovery process that can be described in co‐writing are conceived of as complex, multidimensional and ever‐changing.

Third, these four paradigms follow an *ecological* approach. In this respect, co‐writing is necessarily embedded within a relational and intersubjective space where different *truths* are situated and negotiated.

Beyond these common features, each of these paradigms focuses on specific aspects to address co‐writing. Ethnography values the clinical encounter as a sort of meeting of cultures. Just as the anthropologist's work is aimed at exploring and elucidating different features of novel socio‐cultural worlds, clinicians who want to support co‐writing should explore the initially unknown and novel territory of the client's experience and narrative with great respect and honesty. As ethnographers, clinicians should be aware of how they can bring their subjectivity, views, perspectives, politics and passions into facilitating co‐writing, as well as promoting a critical reflection on their role in the co‐production of knowledge.

Value‐based practice strongly opposes coercive instruments, especially in the case of some psychiatric practices, including the unequal power relationship between the doctor as a provider of treatment and the patient as the person receiving it. In this framework, co‐writing is conceived of as a practice of co‐production, based on a raised awareness of the role of values even in categorical psychiatric diagnostic systems.

The narrative approach emphasizes the therapeutic role of expressive writing about the self. The storytelling process includes symptoms as part of a meaningful experience and the story as a tool for potential healing. In this context, co‐writing allows for the exploration of preferences, needs and hopes as sedimentations of personal histories and as modalities that give meaning to and frame experiences. Moreover, this joint exploration may improve the clinician–client relationship.

Phenomenology devotes its focus to the study of lived experience, enabling a rich conception of the co‐writing process in its aspects of self‐awareness, embodiment, spatiality and intersubjectivity. If this approach emphasizes the importance of recognizing the *existential difference*—the individual autonomy of the patient—that separates the clinician's perspective from the way of being in the world that characterizes the former, at the same time, through the bracketing of both our obvious/common sense and our theoretical/scientific pre‐knowledge, it stresses the importance of the concept of dialogue, highlighting the importance of mutual recognition within the clinical encounter (Orange, 2010). Moreover, after having explored and given new meanings to pre‐reflective subjective experiences, phenomenology aims at exploring clients’ life histories (their specific *world projects*) as *existential a priori*, along with the *themes* upon which they are grounded.

In conclusion, the four paradigms presented in this paper are based on a more global, holistic way of considering lived experience, using specific modalities and linguistic devices to give meaning to individual existence with all its potential mental symptoms, beyond and apart from their eventual biological basis. They provide a ground and rationale for co‐writing as a new response to patients’ dilemmas in the mental healthcare system.

## CONFLICT OF INTEREST

The authors declared no conflict of interest.

## AUTHOR CONTRIBUTIONS

The design of the manuscript was made by the first author (EF), and it has been supervised and completed by the last author (GS). The second (RP) and the third authors (RV) made comments and wrote the conclusions. GS also conceived the idea at the base of this paper.

## ETHICAL APPROVAL

This paper is based on literature review. It does not need an academic ethics committee approval. It was conceptualized for the section: Essays and Debates in Mental Health.

## Data Availability

This is a debate essay, no data was analyzed.
